# Massive Biventricular Thrombi in a Patient with Endometrial Adenocarcinoma Diagnosed on Computed Tomography Angiography

**DOI:** 10.1177/11795468231182762

**Published:** 2023-06-23

**Authors:** Shiva Barforoshi, Justin Sharim, Matthew J Budoff

**Affiliations:** 1 Department of Medicine, Harbor-UCLA Medical Center, Torance, CA, USA; 2 Department of Cardiology, Harbor-UCLA Medical Center, Torrance, CA, USA; 3 Lundquist Institute, Harbor-UCLA Medical Center, Torrance, CA, USA

**Keywords:** Biventricular thrombi, intracardiac thrombus, computed tomography angiography, embolism, malignancy

## Abstract

Biventricular thrombi are a rare clinical entity and only reported in several case reports. Given ventricular thrombi are high risk for cardioembolic events, accurate detection and therapeutic management has an important influence on clinical outcomes. We present a case of a patient with biventricular thrombi that was initially diagnosed on computed tomography angiography, emphasizing its clinical utility as a rapid, non-invasive imaging modality for early detection.

## Introduction

Biventricular thrombi are relatively rare, even in clinical conditions with a high propensity for thrombus formation including malignancy, antiphospholipid antibody syndrome, and heparin-induced thrombocytopenia. Only a small number of prior case reports have reported biventricular thrombi.^1-7^ Given that ventricular thrombi are high risk for cardioembolic events, accurate detection and therapeutic management has an important influence on clinical outcomes. We present a case report of a patient with biventricular thrombi detected on computed tomography angiography (CTA) imaging, highlighting its utility as a non-invasive diagnostic tool in early detection of intracardiac thrombi.

## Case

A 60-year-old woman with a history of heart failure with reduced ejection fraction of 20% to 25% secondary to non-ischemic cardiomyopathy, hypertension, type 2 diabetes complicated by chronic kidney disease, remote history of stroke with residual right sided weakness, and stage IVB grade 2 endometrial adenocarcinoma with omental metastasis post hysterectomy/salpinoophorectomy presented with intermittent, pleuritic, chest pain in the middle of her chest radiating to her back and shortness of breath with exertion. She had nuclear perfusion imaging completed 3 months prior to admission with no evidence of ischemia or infarct. She endorsed being compliant with her heart failure medications (furosemide 40 mg/day, metoprolol succinate 25 mg/day). She recently received adjuvant chemotherapy 1 month prior. On arrival, she was found to be hypotensive with systolic blood pressure of 90 mmHg, but otherwise clinically stable. On exam she had no cardiac murmurs, rubs, or gallops, no lower extremity edema, and her lungs were clear to auscultation bilaterally. Pertinent initial laboratory workup revealed the following findings: troponin I 0.158 ng/mL (normal range, 0.02-0.03 ng/mL), absolute neutrophil count of 0, white blood cell count 0.05 K/μL (normal range, 3.70-10.40 K/μL), hemoglobin of 9.2 g/dL, beta natriuretic peptide of 681 pg/mL (normal range, 5.0-100.0 pg/mL).

**Figure 1.**
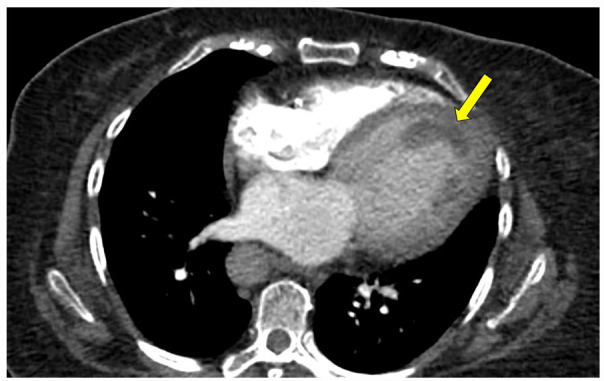
CTPA, axial dual-chamber view, contrast-enhanced. Arrow indicates mass within the left ventricle with characteristics consistent with thrombus.

**Figure 2.**
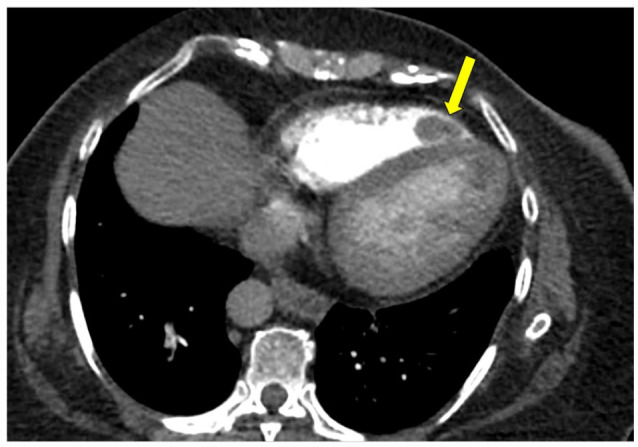
CTPA, axial dual-chamber view, contrast-enhanced. Arrow indicates mass within the right ventricle with characteristics consistent with thrombus.

**Figure 3.**
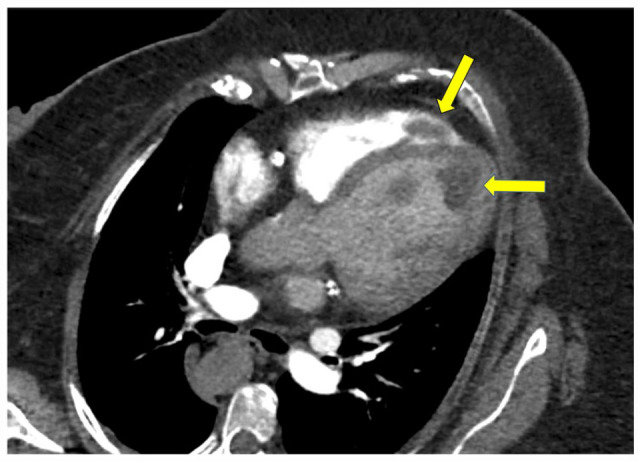
CTPA, oblique 4-chamber view, contrast-enhanced. Arrows indicated masses with characteristics consistent with thrombi.

**Figure 4.**
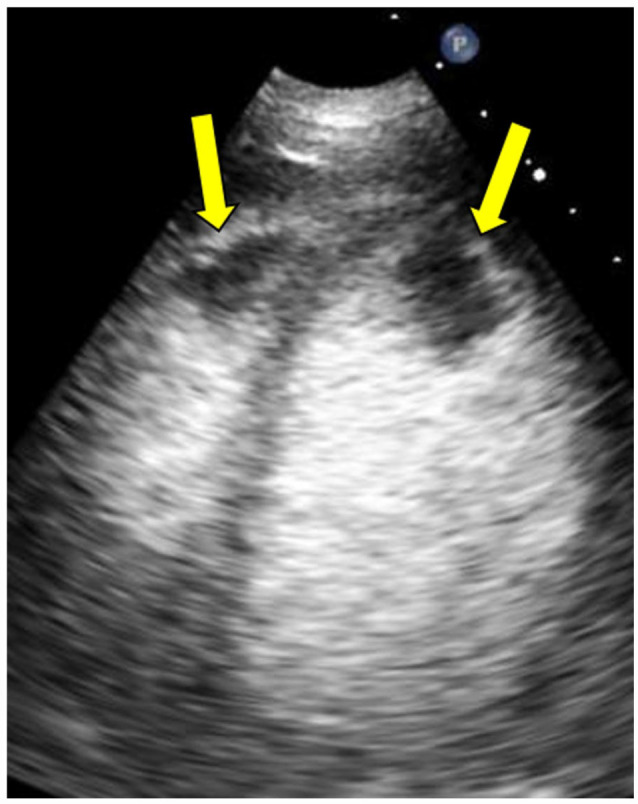
Transthoracic echocardiography with definity contrast, apical 4-chamber view. Arrows indicate large apical thrombi in the right and left ventricles.

**Figure 5.**
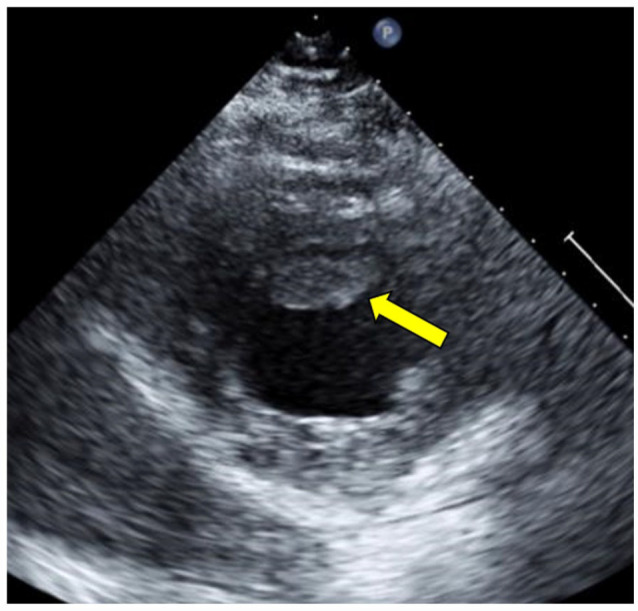
Transthoracic echocardiography with definity contrast, parasternal long view. Arrow indicates large, mobile apical thrombi in the left ventricle.

Chest radiography revealed cardiomegaly. Electrocardiography revealed sinus tachycardia, left bundle branch block, and left ventricular hypertrophy that were stable compared to prior. She underwent computed tomography angiography pulmonary angiogram (CTPA) to rule out pulmonary embolism which revealed filling defects of the apex of the right and left cardiac ventricles (Figures 1-3), concerning for biventricular thrombi; negative for pulmonary embolism. Transthoracic echocardiography (TTE) revealed a left ventricular ejection fraction of 20% to 25% and biventricular thrombi (Figures 4 and 5). The left ventricle had a large, mobile thrombus measuring up to 4.6 cm × 1.5 cm and the right ventricular thrombus was 2.6 cm in diameter. These thrombi were new findings compared with TTE obtained 3 months prior. Surgical intervention via thrombectomy to remove ventricular clot burden was discussed given high risk for embolization, however following multidisciplinary discussion, the patient was deemed too high risk for surgery given her multiple comorbidities. CT head revealed new subacute infarct and follow up MRI brain imaging confirmed acute left parietal infarct and subacute to chronic right occipital infarct. Since she was not a good surgical candidate for thrombectomy, medical management with anticoagulation was pursued. Neurology deemed it safe to initiate a heparin drip with serial head imaging given both intracranial infarcts were small with low risk of hemorrhagic conversion. Her clinical course was complicated by concern for heparin induced thrombocytopenia (HIT) and was therefore she was transitioned to argatroban drip. HIT was later ruled out with negative serotonin release assay and HIT ELISA, and the patient was transitioned back to heparin drip and bridged to warfarin for INR goal of 2-3. The patient was ultimately discharged with warfarin with plan for indefinite anticoagulation in the setting of her ongoing malignancy.

## Discussion

The occurrence of biventricular thrombi is a rare but serious condition which may increase the risk of both pulmonary and systemic embolization. Only few cases have been previously reported, and it has been described in association with severe biventricular dysfunction,^8^ hypercoaguable states,^2^ infection (including COVID-19),^9^ autoimmune disease,^1^ drug-related,^5^ and myocardial infarction.^3^ We believe the etiology of her biventricular thrombi may have been multifactorial from her dilated cardiomyopathy likely from longstanding heart failure along with her hypercoagulable state given her underlying malignancy. Tumor metastasis to the heart was considered, however is rare with endometrial adenocarcinoma.^10^ While she underwent CTPA for her chest pain and shortness of breath to rule out pulmonary embolism, it incidentally revealed biventricular thrombi. Furthermore, both pulmonary embolism and deep vein thromboses were ruled out, indicating isolated intracardiac thrombi.

Ventricular thrombus is a potentially life-threatening condition as it puts patients at an increased risk for embolic complications, including pulmonary embolism or stroke^4^ (as seen in our patient). Therefore, understanding imaging modalities for early detection of ventricular thrombi carries clinical significance in preventing its potential complications. While there is a plethora of imaging modalities to diagnose intracardiac thrombi, including TTE, transesophageal echocardiography (TEE), CTA, and Cardiac magnetic resonance imaging, CT is an emerging imaging modality with high diagnostic performance for detection of intracardiac thrombi. Detection of ventricular thrombi is generally performed by TTE while atrial thrombi are generally evaluated by TEE, both of which are optimized by a contrast-enhancing agent. CTA, however, has the advantage of being more sensitive for detecting both atrial and ventricular thrombi than TTE but inferior for displaying atrial thrombi compared to TEE.^11,12^ While TEE has been the gold standard for left atrial and left atrial appendage thrombi detection, CCTA has mean sensitivity and specificity of 96% and 92%, respectively, serving as a reliable alternative imaging modality, while avoiding the invasiveness, discomfort, and risks associated with TEE. Additionally, while TTE is generally performed for left ventricular thrombi, it has lowest sensitivity (10%) for detection of thrombi less than 10 mm, and highest sensitivity for thrombi greater than 20 mm due to the limited near field resolution and resemblance of mural thrombi to adjacent myocardium. Furthermore, both TTE and TEE are both operator dependent techniques and influenced by individual patient characteristics such as chest wall anatomy or esophageal disease, respectively, which can be avoided in CTA imaging.^13,14^

Overall, while TTE is sensitive for ventricular thrombi and TEE is sensitive for atrial thrombi, CTA serves as a more comprehensive imaging modality, addressing the diagnostic challenges of both the others modalities as mentioned above. This case highlights the clinical utility of CTA as a single high yield, non-invasive imaging modality not only for detection of intracardiac thrombi but also in evaluating for multiple diagnoses at once. In our patient, CTA successfully detected biventricular thrombi and simultaneously ruled out pulmonary embolism, allowing for initiation of early anticoagulation and guiding the differential diagnosis for her decompensation. CTA imaging has great clinical utility in being rapid and non-invasive, allowing for timely diagnosis and management of intracardiac thrombi.

## Learning Objectives

1. To understand the differential diagnoses for etiology of biventricular thrombi and its complications.
2. To value the clinical utility of computed tomography angiography imaging as a rapid, non-invasive imaging modality for detection of intracardiac thrombi.
